# *metagene* Profiles Analyses Reveal Regulatory Element’s Factor-Specific Recruitment Patterns

**DOI:** 10.1371/journal.pcbi.1004751

**Published:** 2016-08-18

**Authors:** Charles Joly Beauparlant, Fabien C. Lamaze, Astrid Deschênes, Rawane Samb, Audrey Lemaçon, Pascal Belleau, Steve Bilodeau, Arnaud Droit

**Affiliations:** 1 Centre de Recherche du CHU de Québec - Université Laval, Québec, Québec, Canada; 2 Département de Médecine Moléculaire, Faculté de médecine, Québec, Canada; 3 Centre de Recherche sur le Cancer de l’Université Laval, Québec, Québec, Canada; 4 Département de Biologie Moléculaire, Biochimie Médicale et Pathologie, Faculté de médecine, Québec, Canada; University of Canterbury, NEW ZEALAND

## Abstract

ChIP-Sequencing (ChIP-Seq) provides a vast amount of information regarding the localization of proteins across the genome. The aggregation of ChIP-Seq enrichment signal in a metagene plot is an approach commonly used to summarize data complexity and to obtain a high level visual representation of the general occupancy pattern of a protein. Here we present the R package *metagene*, the graphical interface *Imetagene* and the companion package *similaRpeak*. Together, they provide a framework to integrate, summarize and compare the ChIP-Seq enrichment signal from complex experimental designs. Those packages identify and quantify similarities or dissimilarities in patterns between large numbers of ChIP-Seq profiles. We used *metagene* to investigate the differential occupancy of regulatory factors at noncoding regulatory regions (promoters and enhancers) in relation to transcriptional activity in GM12878 B-lymphocytes. The relationships between occupancy patterns and transcriptional activity suggest two different mechanisms of action for transcriptional control: i) a “gradient effect” where the regulatory factor occupancy levels follow transcription and ii) a “threshold effect” where the regulatory factor occupancy levels max out prior to reaching maximal transcription. *metagene*, *Imetagene* and *similaRpeak* are implemented in R under the Artistic license 2.0 and are available on Bioconductor.

This is a *PLOS Computational Biology* Software paper.

## Introduction

Understanding the global regulation of gene expression programs is an important goal of functional genomics studies. To this end, it is now standard procedure to survey the occupancy of regulatory proteins genome-wide using chromatin immunoprecipitation coupled with massively parallel sequencing (ChIP-Seq) [1]. Affordability and accessibility of the technique are now generating more complex experimental designs containing many samples, treatments, controls comparisons and technical replicates. Furthermore, the abundance of public datasets, such as those provided by the ENCODE [2] and Roadmap Epigenomics [3] consortiums, provides a wealth of information. Unfortunately, the integration of large amounts of ChIP-Seq information remains challenging.

In a typical ChIP-Seq analysis, reads are first aligned using an aligner of choice and peaks are called using peak calling algorithms, such as MACS [4] or PICS [5], to obtain a list of occupied regions. Then, these regions are annotated to genes [6] and/or used to search for DNA binding motifs [7]. In addition, tools were developed to quantitatively compare regions from ChIP-Seq experiments in order to define regions with differential binding between conditions [8]. The algorithms and models used to manage background, to normalize read counts and to estimate the reads distribution across the genome are the main differences between the different methods. While these tools allow the discovery of regions that are differentially occupied by a factor of interest, they are unable to evaluate differences in the general occupancy patterns of DNA-binding proteins. Furthermore, they rely on the peak calling step which varies greatly based on the algorithm or the parameters used [9].

Current approaches to compare and summarize enrichment signals for groups of regions rely on visual representations of the average enrichment at a specific position. These representations are known as metagene plots (also referred to as meta-gene [10] or aggregation plots [11]). To compare multiple samples, many tools implemented reads per million aligned [11, 12] or quantile [13, 14] normalizations. The addition of confidence intervals (represented as ribbons) based on standard errors (of mean or of percentiles) in *ngs.plot* [12], on bootstrap approaches in *ChIPseeker* [15] or as standard error in *seqPlots* [13] improved the prediction of the mean. However, while confidence intervals are effective tools to estimate the range within which the true mean is likely to lie, profile comparisons require statistical testing. In addition, valuable information embedded in the enrichment profiles such as the position of the binding event inside the region or the presence of a specific pattern notwithstanding its amplitude is currently ignored. Therefore, representation tools enabling a quantitative assessment and robust statistical comparisons of metagene profiles are needed.

We developed the *metagene* package to quantitatively compare enrichment profiles of group of regions. Specifically, this package is designed to 1) facilitate the integration of signal from many datasets linked by complex experimental designs, 2) statistically compare the enrichment profiles of groups of genomic regions and 3) provide visual representations of the data to facilitate interpretation. Here we used the *metagene* package to investigate how regulatory factors contribute to the transcriptional output of noncoding regulatory regions. Indeed, recruitment of regulatory factors to noncoding regulatory regions, including enhancer and promoter regions, modulates the transcriptional response of each gene. Using the *metagene* and *similaRpeak* package, we identified the similarities and dissimilarities in the recruitment patterns of these factors at enhancer and promoter regions. Our results demonstrate that there are two distinct mechanisms of action for transcriptional regulators. Indeed, we discovered that the level of the regulatory factors either correlates with the transcriptional activity or saturates prior to maximal transcriptional activity of the regulatory region. We termed those patterns “gradient effect” and “threshold effect”.

## Design and Implementation

**Fig 1:**
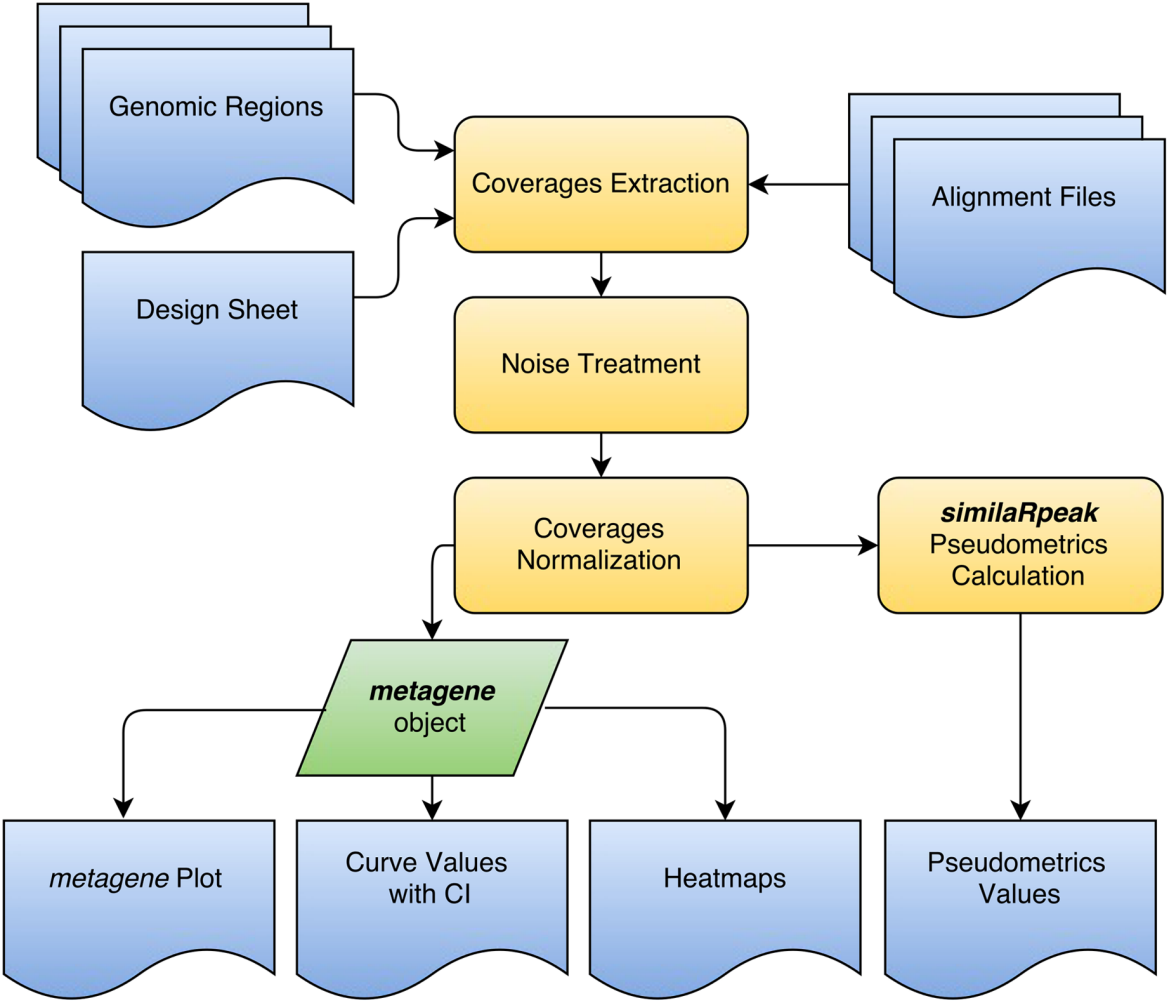
*metagene* workflow. A *metagene* analysis requires 3 types of inputs: 1) a list of genomic regions (BED or GRanges formats), 2) alignment files (BAM format) and 3) a design sheet (data frame format) explaining the relations between samples. The alignment files are processed to extract the coverages of every genomic regions. Afterward, the background is removed from the coverages and the signal is normalized (reads per millions aligned or RPM) to allow comparison between samples. The main output is the metagene plot. The other outputs are the curve values and confidence intervals (CI) used to produce the plot and an interactive heatmap with *Imetagene*. The results are compatible with *similaRpeak* for profile characterization.

The *metagene* package builds upon Bioconductor scalable data structures for representing annotated ranges on the genome [16]. Additionally, to efficiently import large datasets, *metagene* supports the most common genomic file formats such as bam, bed and narrowPeak/broadPeak. The number of files used in a single analysis is only limited by the computer memory available. To reduce memory usage, *metagene* produces coverages only for the genomic regions of interest and stores this information in Run-length encoding. It is possible to compare multiple region groups and multiple experiments in a single analysis. To increase the analytical power, *metagene* uses the controls to estimate the signal-to-noise ratio and remove background signal. The datasets are also normalized for an accurate comparison. Furthermore, the directionality of the genomic regions (i.e. the strand) is usable to highlight asymmetric enrichment patterns. In the final graphical output, the metagene plot, each curve summarizes the information of multiple genomic regions (termed region groups) from a single experiment. When used with the *similaRpeak* package, our approach allows the comparison of multiple samples and gives the possibility to statistically compare the results with metrics adapted to different profile features. The *Imetagene* package offers a simple graphical interface to manage complex experimental designs. A workflow of a typical *metagene* analysis is provided in Fig 1.

**Fig 2:**
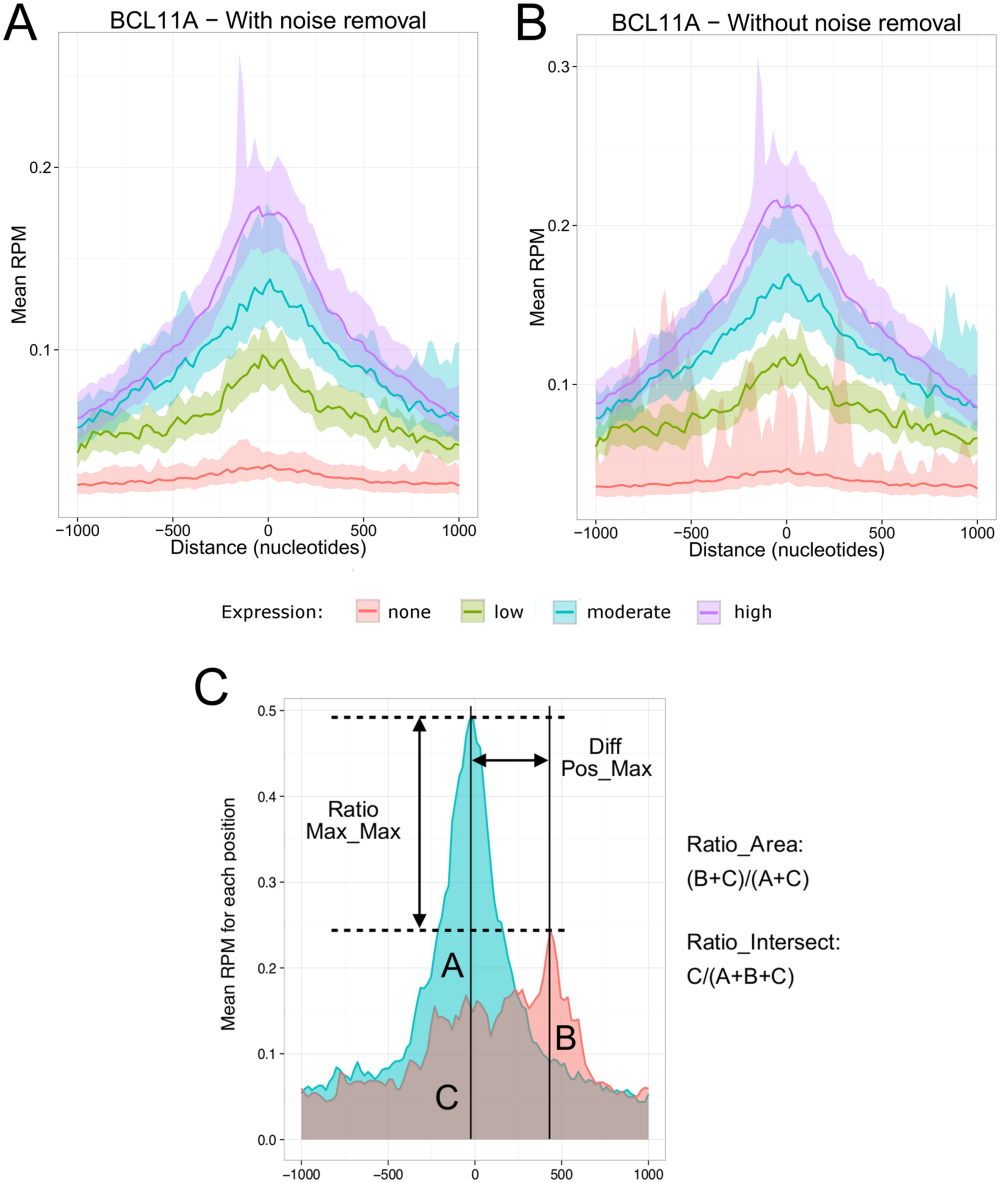
Impact of noise removal and description of the pseudometrics. Metagene plots of the BCL11A transcription factor (A) with noise removal using the NCIS algorithm and (B) without noise removal. The x-axis is centered on enhancers and promoters ±1000bp. The y-axis represents the mean occupancy normalized in reads per million (RPM). Each line represents the mean occupancy of the BCL11A replicates. Groups of transcriptional activity of enhancers or promoters are identified by different colors (red = no CAGE signal; green = low CAGE signal; blue = moderate CAGE signal; purple = high CAGE signal; see S1 Text). Ribbons represent the 95% confidence interval of the mean calculated using 1000 bootstraps. (C) Description of some of the pseudometrics implemented in the *similaRpeak* packages.

In order to quantitatively compare different experiments, it is crucial to take into account the signal-to-noise ratio and to normalize samples. Indeed, the ChIP-Seq signal is a mixture of legitimate signal and noise. The experimental noise is influenced by biological factors such as the GC content and the chromatin structure [17] and by technical factors such as the antibody quality, the cell number, the DNA fragmentation and the library construction [18]. A common approach to separate true signal from noise is to use controls. Ideally, the controls should be normalized to fit only with the noise component of the chip signal since only this part of the signal will follow the same distribution [19]. In order to normalize the controls before subtracting the background, *metagene* uses the Normalization of ChIP-seq (NCIS) approach [20] to calculate the signal to noise ratio. This approach performs well on ChIP-Seq datasets [19] and is readily available in R (Fig 2A and 2B show the effect of noise reduction). If multiple samples are compared together, they should be normalized to take into account the difference in library sizes. This is performed in *metagene* by converting the raw coverage values in read per millions aligned. It is also possible to change the orientation of each genomic region on the negative strand to represent every region in the 5’→3’ orientation. The profile of each group defined in the design is calculated using either an average or median profile, as specified by the user. A confidence intervals of the estimators (mean or median) is computed at each base pair using bootstraps (1000 times by default) for each group profile. To reduce the effects of extreme coverage values, a data binning strategy with customizable bin sizes, is applied before bootstrapping. Visually, the confidence interval is represented by a ribbon which includes an editable percentage (default 95%) of the sampled values (see S1 Text for more information on the bootstrap approach implemented in *metagene*). Using the *Imetagene* package, it is also possible to preview the regions as an interactive heatmap (S1 Fig).

A unique feature of the *metagene* package is the implementation of a statistical comparison between profiles to detect differential enrichment. The comparison is done through a permutation test using metrics which are specified by the user that is not related to the confidence intervals calculated with bootstrapping. For each round of the permutation test, the metric value is calculated using two profiles obtained by randomly sampling the coverages used to compute the original profiles. The proportion of metric scores above the original score is used to calculate a p-value and determine if two profiles are significantly different (see S1 Text for more details). By enabling the use of a diversity of metrics, the statistical comparison can be tailored to fit custom needs. To facilitate the identification of common patterns between two ChIP-Seq profiles, *similaRpeak* is proposed as a companion package to *metagene*. The *similaRpeak* package implements six pseudometrics specialized in pattern similarity detection (Fig 2C). The profile submitted to each pseudometric must respect certain editable criterias, specific to each pseudometric, to ensure that the calculation of the pseudometric is only made in presence of informative peaks and to limit the computation of extreme values. A description of each pseudometric is available in S2 Table. Lastly, we developed a graphical user interface powered by Shiny [21], *Imetagene*. This graphical interface was developed to facilitate the use of *metagene* without R programming experience. Taken together, this set of software is used to quickly compare multiple region groups to discover enrichment patterns that would otherwise be missed when looking at individual regions.

## Results

Proper spatiotemporal transcription requires the complex interplay of transcription factors, cofactors and chromatin regulators at noncoding regulatory regions [22, 23]. Indeed, enhancer and promoter regions recruit regulatory factors to modulate the recruitment, initiation, pause-release and elongation of the RNA polymerase II (Pol II) [24, 25]. During the transcriptional process, both enhancer and promoter regions are transcribed [26–28]. Here we use the *metagene* package to correlate the recruitment of regulatory factors at enhancer and promoter regions with their transcriptional output.

### Data collection and *metagene* analyses

To define the contribution of transcription factors and cofactors to the transcriptional activity of promoters and enhancers, we gathered the publically available data generated in GM12878 B-lymphocytes (106 available experiment datasets; 276 alignment files, information in S1 Table). Promoters regions were obtained using the Bioconductor’s TxDb.Hsapiens.UCSC.hg19.knownGene package [16] and enhancers were downloaded from the Fantom5 database [29]. Robust enhancer and promoter regions were defined by regions with at least one robust transcription start site (TSS) in the Fantom5 database. Finally, the regions were stratified into four groups based on their cap analysis of gene expression (CAGE) levels [28]: “no expression”, “low expression”, “moderate expression” and “high expression” (see S1 Text).

### Pol II and the general transcription factors levels correlate with transcriptional activity

**Fig 3:**
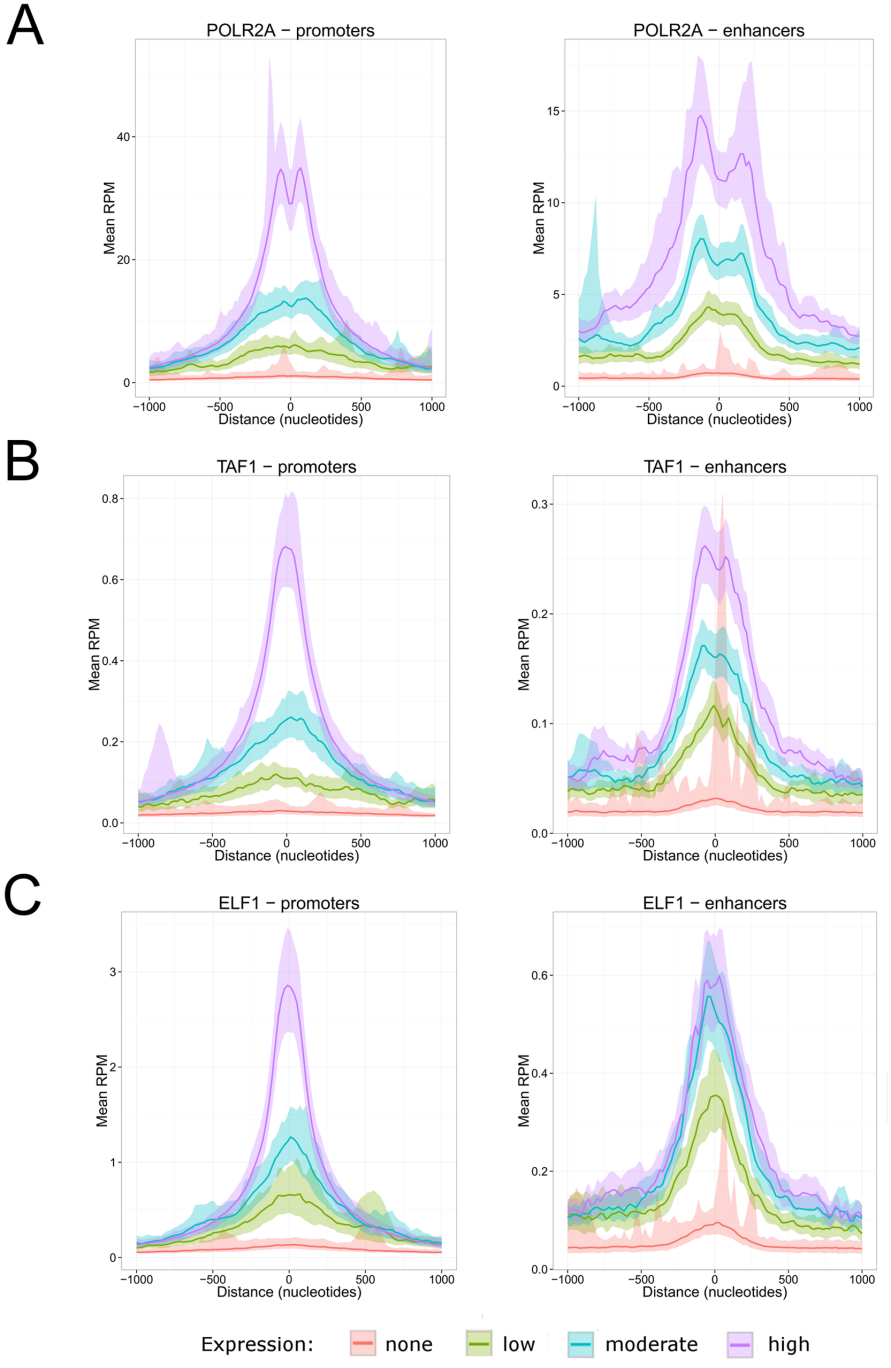
Metagene profiles in enhancer and promoter regions. (A) POLR2A, the largest subunit of Pol II. (B) TAF1, a general transcription factor. (C) ELF1, a transcription factor. The x-axis is centered on enhancers and promoters ±1000bp. The y-axis represents the mean occupancy normalized in reads per million (RPM). Each line represents the mean occupancy of the factor replicates. Groups of transcriptional activity of enhancers or promoters are identified by different colors (red = no CAGE signal; green = low CAGE signal; blue = moderate CAGE signal; purple = high CAGE signal). The ribbons represent the 95% confidence interval of the mean calculated using 1000 bootstraps.

To validate our transcriptional stratification of enhancers and promoters, we surveyed the occupancy of total Pol II and the general transcription factors (GTFs), in function of the transcriptional activity [30–32]. As expected, transcriptional levels of enhancer and promoter regions correlated with recruitment of Pol II (Fig 3A and S2 and S3 Figs), TAF1 (Fig 3B), and TBP (S4 Fig). Histone marks associated with active enhancers (H3K27ac) and with active promoters (H3K4me3) showed a similar pattern (S5 Fig). The RATIO INTERSECT pseudometric, which calculates the ratio of the area under the intersection of two profiles with the total area, was used to compare the coverage between each group (S3 Table). The pseudometric value tends to 1 as the similarity between profiles increases. The statistical analyses confirmed that an increase in transcriptional activity correlates with an increase in the Pol II machinery (permutation p-value <0.001). In addition, the GTFs followed the same correlation with transcriptional activity. These results demonstrate that *metagene* and *similaRpeak* are able to distinguish patterns associated with different levels of transcription activity in a large number of samples by using robust metrics. Together, they offer an excellent tool to investigate the relationship between recruitment of regulatory factors and transcriptional activity.

### Differential recruitment of regulatory factors at promoter and enhancer regions

While Pol II and GTFs activities are directly linked to the transcriptional output, the importance of each individual regulatory factor for the transcription process is not well understood. To assess the quantitative recruitment of transcription factors, cofactors and chromatin regulators at cis-regulatory elements as a function of the transcriptional activity, we evaluated the occupancy of regulatory factors, histone modifications and DNAse hypersensitive sites in GM12878 cells. Interestingly, we observed two distinct recruitment patterns at promoter and enhancer regions. Indeed, a “gradient effect” was observed when the occupancy level of a factor correlated with the transcriptional activity (Fig 3A and 3B) while a “threshold effect” refers to factors reaching a plateau in their occupancy prior to maximal transcriptional activity (Fig 3C). We defined a “threshold effect” as a ratio between the intersection area and the total area of the two profiles (RATIO INTERSECT) superior or equal to 0.85 between the high and moderate CAGE signal group. Overall, 44.6% of factors showed a “threshold effect” at enhancer regions while only 19.8% were observed at promoter regions (S6 Fig; p-value = 0.0048, Welch’s Two Sample t-test). For example, the transcription factor ELF1 levels correlated with the transcriptional activity at promoters regions (RATIO INTERSECT = 0.66), but not at enhancers regions (RATIO INTERSECT = 0.88) (Fig 3C). A total of 35 regulatory factors including IRF3 and IRF4 (involved in interleukin regulation [33, 34]) and cofactors like SMC3 and EP300 (S7 and S8 Figs) were identified with a similar dichotomy (see S3 Table for a complete list). These results highlight a differential requirement of regulatory factors at enhancer and promoter regions in relation to transcriptional activity.

### Threshold versus gradient effects

Differential recruitment of regulatory factors at promoter and enhancer regions raises mechanistic questions. We are proposing different models to explain the “gradient” and “threshold” effects. For the “threshold effect”, mostly observed at enhancer regions, the regulatory factors are potentially working as “on/off” switches. In that model, once a predetermined level is achieved for a specific transcription factor or cofactors, the transcriptional contribution is maximized (Fig 3B, S7 and S8 Figs and S3 Table). Extrapolation of this model suggests that an accumulation of different regulatory factors is required to achieve maximal transcriptional output at enhancer regions. This idea is corroborated by observations of dozens of transcription factors at enhancers regions in mammalian cells [35]. For the “gradient effect” mostly observed at promoter regions, we are considering two models: i) the regulatory factor directly contributes to Pol II transcriptional activity or ii) the “gradient effect” corresponds to the signal accumulation of multiple enhancers connecting to a promoter region through long distance interactions. These models are not mutually exclusive, but the latter is supported by evidence of an average of 4.9 enhancers connecting per promoter [28] in addition to a positive correlation between the number of connections and the transcriptional output [36]. Taken together, our results establish different recruitment patterns of regulatory factors at enhancers and promoters.

### Other applications of *metagene*

In addition to the current study, the *metagene* package will be usable for multiple applications. For instance, the *metagene* package will be suitable to study differential recruitment in different classes of regulatory elements. For instance, enhancers and promoters regions could be stratified by functional types instead of expressions levels, such as the chromatin states [37]. The enrichment patterns of a transcription factor following drug treatment or an infection could also be analyzed with *metagene* to provide molecular insights into the mechanism of action. Additionally, the dynamic of transcription factors recruitment could be studied using time course datasets. Future studies will reveal new details on the mechanisms of recruitment of regulatory factors and will help in understanding the similarities and dissimilarities between the various classes of regulatory elements.

## Availability and Future Directions

The *metagene* package, the graphical interface *Imetagene*, and the companion package *similaRpeak* are available on Bioconductor with documentation and an example dataset. These packages perform a thorough evaluation of the similarities or dissimilarities of the aggregated signal of region groups. For the current version, the region groups are based on annotations in order to test specific scientific hypotheses. Next, we will work on refinement to the bootstrapping strategy and we will be implementing clustering algorithms (as a part of a machine learning strategy) to cluster regions based directly on their occupancy patterns to provide an exploratory approach.

## Supporting Information

S1 Fig *Imetagene* interactive heatmap representation. After the matrices are computed, the *Imetagene* package can be used to explore the matrix-associated with each experiment to visualize the coverages of the regions.
(PDF)

S2 Fig Metagene plots of RNA Pol II phosphorylated at serine 2 (POLR2AphosphoS2) in promoters and enhancers. The x-axis is centered on enhancers and promoters ±1000bp. The y-axis represents the mean occupancy normalized in reads per million (RPM). Each line represents the mean occupancy of POLR2Aphosphos2. Groups of transcriptional activity of enhancers or promoters are identified by different colors (red = no CAGE signal; green = low CAGE signal; blue = moderate CAGE signal; purple = high CAGE signal; see S1 Text). Ribbons represent the 95% confidence interval of the mean calculated using 1000 bootstraps.
(PDF)

S3 Fig Metagene plots of RNA Pol II phosphorylated at serine 5 (POLR2AphosphoS5) in promoters and enhancers. The x-axis is centered on enhancers and promoters ±1000bp. The y-axis represents the mean occupancy normalized in reads per million (RPM). Each line represents the mean occupancy of POLR2Aphosphos5. Groups of transcriptional activity of enhancers or promoters are identified by different colors (red = no CAGE signal; green = low CAGE signal; blue = moderate CAGE signal; purple = high CAGE signal; see S1 Text). Ribbons represent the 95% confidence interval of the mean calculated using 1000 bootstraps.
(PDF)

S4 Fig Metagene plots of the general transcription factor TBP at promoters and enhancers. The x-axis is centered on enhancers and promoters ±1000bp. The y-axis represents the mean occupancy normalized in reads per million (RPM). Each line represents the mean occupancy of TBP. Groups of transcriptional activity of enhancers or promoters are identified by different colors (red = no CAGE signal; green = low CAGE signal; blue = moderate CAGE signal; purple = high CAGE signal). The ribbons represent the 95% confidence interval of the mean calculated using 1000 bootstraps.
(PDF)

S5 Fig Metagene plots of H3K27ac at enhancers and H3K4me3 at promoters. The x-axis is centered on enhancers and promoters ±1000bp. The y-axis represents the mean occupancy normalized in reads per million (RPM). Each line represents the mean occupancy of the histone mark. Groups of transcriptional activity of enhancers or promoters are identified by different colors (red = no CAGE signal; green = low CAGE signal; blue = moderate CAGE signal; purple = high CAGE signal). The ribbons represent the 95% confidence interval of the mean calculated using 1000 bootstraps.
(PDF)

S6 Fig Boxplot of RATIO INTERSECT values for 106 experiments in GM12878. The RATIO INTERSECT was calculated using the moderate CAGE signal and high CAGE signal groups.
(PDF)

S7 Fig Metagene plots of the cofactor SMC3 at promoters and enhancers. The x-axis is centered on enhancers and promoters ±1000bp. The y-axis represents the mean occupancy normalized in reads per million (RPM). Each line represents the mean occupancy of SMC3. Groups of transcriptional activity of enhancers or promoters are identified by different colors (red = no CAGE signal; green = low CAGE signal; blue = moderate CAGE signal; purple = high CAGE signal). The ribbons represent the 95% confidence interval of the mean calculated using 1000 bootstraps.
(PDF)

S8 Fig Metagene plots of the cofactor EP300 at promoters and enhancers. The x-axis is centered on enhancers and promoters ±1000bp. The y-axis represents the mean occupancy normalized in reads per million (RPM). Each line represents the mean occupancy of EP300. Groups of transcriptional activity of enhancers or promoters are identified by different colors (red = no CAGE signal; green = low CAGE signal; blue = moderate CAGE signal; purple = high CAGE signal). The ribbons represent the 95% confidence interval of the mean calculated using 1000 bootstraps.
(PDF)

S1 Table Description of the 276 bam files used in this article. Experiment accession: unique identifier of the experiment. File accession: unique identifier of the file. Target: the name of the factor that was targeted for immunoprecipitation. Controls: the experiment accession of the recommended controls. Biosample name: the cell type. Assembly: the version of the genome used for the alignment. Href: the URL to download the file.
(CSV)

S2 Table Description of *similaRpeak*’s pseudometrics. Pseudometric: the name of the pseudometric. Definition: the description of the metric. Threshold: criteria that can be set by the user to avoid calculating the value of a pseudometric that would return nonsensical results (division by zero, etc…).
(XLSX)

S3 Table Classification of GM12878 factors. The classification of the 106 regulatory factors in “gradient” or “threshold”. Target: the name of the target. Type: enhancer or promoter. RATIO_INTERSECT: the RATIO_INTERSECT score calculated using the moderate and high CAGE signal groups. Class: “gradient” or “threshold”.
(CSV)

S1 Text Data collection: Details of the data collection procedure. Bootstrap: Description of the bootstrapping steps. Permutation: Details of the permutation procedure in *Metagene* and *similaRpeak*.
(PDF)

## References

[1] Johnson DS, Mortazavi A, Myers RM, Wold B. Genome-Wide Mapping of in Vivo Protein-DNA Interactions. Science. 2007;316(5830):1497–1502. 10.1126/science.1141319 17540862

[2] Dunham I, Kundaje A, Aldred SF, Collins PJ, Davis CA, Doyle F, et al An integrated encyclopedia of DNA elements in the human genome. Nature. 2012;489(7414):57–74. 10.1038/nature11247 22955616 PMC3439153

[3] Bernstein BE, Stamatoyannopoulos JA, Costello JF, Ren B, Milosavljevic A, Meissner A, et al The NIH Roadmap Epigenomics Mapping Consortium. Nature Biotechnology. 2010;28(10):1045–1048. 10.1038/nbt1010-1045 20944595 PMC3607281

[4] Zhang Y, Liu T, Meyer Ca, Eeckhoute J, Johnson DS, Bernstein BE, et al Model-based Analysis of ChIP-Seq (MACS). Genome Biology. 2008;9(9):R137 10.1186/gb-2008-9-9-r137 18798982 PMC2592715

[5] Zhang X, Robertson G, Krzywinski M, Ning K, Droit A, Jones S, et al PICS: Probabilistic Inference for ChIP-seq. Biometrics. 2011;67(1):151–163. 10.1111/j.1541-0420.2010.01441.x 20528864

[6] Zhu LJ, Gazin C, Lawson ND, Pagès H, Lin SM, Lapointe DS, et al ChIPpeakAnno: a Bioconductor package to annotate ChIP-seq and ChIP-chip data. BMC Bioinformatics. 2010;11(1):237 10.1186/1471-2105-11-237 20459804 PMC3098059

[7] Bailey TL, Williams N, Misleh C, Li WW. MEME: discovering and analyzing DNA and protein sequence motifs. Nucleic Acids Research. 2006;34(Web Server):W369–W373. 10.1093/nar/gkl198 16845028 PMC1538909

[8] Wu DY, Bittencourt D, Stallcup MR, Siegmund KD. Identifying differential transcription factor binding in ChIP-seq. Frontiers in Genetics. 2015;6(April):1–11. 25972895 10.3389/fgene.2015.00169 PMC4413818

[9] Szalkowski AM, Schmid CD. Rapid innovation in ChIP-seq peak-calling algorithms is outdistancing benchmarking efforts. Briefings in Bioinformatics. 2011;12(6):626–633. 10.1093/bib/bbq068 21059603

[10] Shin H, Liu T, Manrai AK, Liu XS. CEAS: cis-regulatory element annotation system. Bioinformatics. 2009;25(19):2605–2606. 10.1093/bioinformatics/btp479 19689956

[11] Kundaje A, Kyriazopoulou-Panagiotopoulou S, Libbrecht M, Smith CL, Raha D, Winters EE, et al Ubiquitous heterogeneity and asymmetry of the chromatin environment at regulatory elements. Genome Research. 2012;22(9):1735–1747. 10.1101/gr.136366.111 22955985 PMC3431490

[12] Shen L, Shao N, Liu X, Nestler E. ngs.plot: Quick mining and visualization of next-generation sequencing data by integrating genomic databases. BMC Genomics. 2014;15(1):284 10.1186/1471-2164-15-284 24735413 PMC4028082

[13] Stempor P. seqplots: An interactive tool for visualizing NGS signals and sequence motif densities along genomic features using average plots and heatmaps; 2014. Available from: http://github.com/przemol/seqplots.

[14] Dharmalingam G, Carroll T. soGGi: Visualise ChIP-seq, MNase-seq and motif occurrence as aggregate plots Summarised Over Grouped Genomic Intervals; 2015.

[15] Yu G, Wang LG, He QY. ChIPseeker: an R/Bioconductor package for ChIP peak annotation, comparison and visualization. Bioinformatics. 2015;31(14):2382–2383. 10.1093/bioinformatics/btv145 25765347

[16] Lawrence M, Huber W, Pagès H, Aboyoun P, Carlson M, Gentleman R, et al Software for Computing and Annotating Genomic Ranges. PLoS Computational Biology. 2013;9(8):e1003118 10.1371/journal.pcbi.1003118 23950696 PMC3738458

[17] Kuan PF, Chung D, Pan G, Thomson JA, Stewart R, Keleş S. A Statistical Framework for the Analysis of ChIP-Seq Data. Journal of the American Statistical Association. 2011;106(495):891–903. 10.1198/jasa.2011.ap09706 26478641 PMC4608541

[18] Kidder BL, Hu G, Zhao K. ChIP-Seq: technical considerations for obtaining high-quality data. Nature immunology. 2011;12(10):918–22. 10.1038/ni.2117 21934668 PMC3541830

[19] Angelini C, Heller R, Volkinshtein R, Yekutieli D. Is this the right normalization? A diagnostic tool for ChIP-seq normalization. BMC Bioinformatics. 2015;16(1):150 10.1186/s12859-015-0579-z 25957089 PMC4448883

[20] Liang K, Keleş S. Normalization of ChIP-seq data with control. BMC bioinformatics. 2012;13:199 10.1186/1471-2105-13-199 22883957 PMC3475056

[21] Chang W, Cheng J, Allaire J, Xie Y, McPherson J. shiny: Web Application Framework for R; 2016. Available from: https://CRAN.R-project.org/package=shiny.

[22] Ong C, Corces V. Enhancer function: new insights into the regulation of tissue-specific gene expression. Nature Reviews Genetics. 2011;12(4):283–93. 10.1038/nrg2957 21358745 PMC3175006

[23] Vernimmen D, Bickmore WA. The Hierarchy of Transcriptional Activation: From Enhancer to Promoter. Trends in Genetics. 2015;31(12):696–708. 10.1016/j.tig.2015.10.004 26599498

[24] Lenhard B, Sandelin A, Carninci P. Metazoan promoters: emerging characteristics and insights into transcriptional regulation. Nat Rev Genet. 2012;13(4):233–245. 22392219 10.1038/nrg3163

[25] Plank JL, Dean A. Enhancer function: Mechanistic and genome-wide insights come together. Molecular Cell. 2014;55(1):5–14. 10.1016/j.molcel.2014.06.015 24996062 PMC8441652

[26] Kim TK, Hemberg M, Gray JM, Costa AM, Bear DM, Wu J, et al Widespread transcription at neuronal activity-regulated enhancers. Nature. 2010;465(7295):182–7. 10.1038/nature09033 20393465 PMC3020079

[27] de Santa F, Barozzi I, Mietton F, Ghisletti S, Polletti S, Tusi BK, et al A large fraction of extragenic RNA Pol II transcription sites overlap enhancers. PLoS Biology. 2010;8(5). 10.1371/journal.pbio.1000384 20485488 PMC2867938

[28] Andersson R, Gebhard C, Miguel-Escalada I, Hoof I, Bornholdt J, Boyd M, et al An atlas of active enhancers across human cell types and tissues. Nature. 2014;507(7493):455–461. 10.1038/nature12787 24670763 PMC5215096

[29] Lizio M, Harshbarger J, Shimoji H, Severin J, Kasukawa T, Sahin S, et al Gateways to the FANTOM5 promoter level mammalian expression atlas. Genome Biology. 2015;16(1):22 10.1186/s13059-014-0560-6 25723102 PMC4310165

[30] Sainsbury S, Bernecky C, Cramer P. Structural basis of transcription initiation by RNA polymerase II. Nature reviews Molecular cell biology. 2015;16(3):129–143. 10.1038/nrm3952 25693126

[31] Murakami K, Elmlund H, Kalisman N, Bushnell DA, Adams CM, Azubel M, et al Architecture of an RNA Polymerase II Transcription Pre-Initiation Complex. Science. 2013;342(6159):1238724–1238724. 10.1126/science.1238724 24072820 PMC4039082

[32] Roeder RG. Role of General and Gene-specific Cofactors in the Regulation of Eukaryotic Transcription. Cold Spring Harbor Symposia on Quantitative Biology. 1998;63:201–218. 10.1101/sqb.1998.63.201 10384284

[33] Fitzgerald KA, McWhirter SM, Faia KL, Rowe DC, Latz E, Golenbock DT, et al IKK*ϵ* and TBK1 are essential components of the IRF3 signaling pathway. Nature Immunology. 2003;4(5):491–496. 10.1038/ni921 12692549

[34] Rengarajan J. Interferon Regulatory Factor 4 (IRF4) Interacts with NFATc2 to Modulate Interleukin 4 Gene Expression. Journal of Experimental Medicine. 2002;195(8):1003–1012. 10.1084/jem.20011128 11956291 PMC2193700

[35] Arnosti DN, Kulkarni MM. Transcriptional enhancers: Intelligent enhanceosomes or flexible billboards? Journal of Cellular Biochemistry. 2005;94(5):890–898. 10.1002/jcb.20352 15696541

[36] Schoenfelder S, Furlan-Magaril M, Mifsud B, Tavares-Cadete F, Sugar R, Javierre BM, et al The pluripotent regulatory circuitry connecting promoters to their long-range interacting elements. Genome Research. 2015;25(4):582–597. 10.1101/gr.185272.114 25752748 PMC4381529

[37] Kundaje A, Meuleman W, Ernst J, Bilenky M, Yen A, Heravi-Moussavi A, et al Integrative analysis of 111 reference human epigenomes. Nature. 2015;518(7539):317–330. 10.1038/nature14248 25693563 PMC4530010

