# Supplementary material for: metagene Profiles Analyses Reveal Regulatory Element’s Factor-Specific Recruitment Patterns
Source: PLoS Comput Biol. 2016 Aug 18;12(8):e1004751. doi: 10.1371/journal.pcbi.1004751 (PMC4990179; doi:10.1371/journal.pcbi.1004751)

S1 Fig. lmetagene interactive heatmap representation

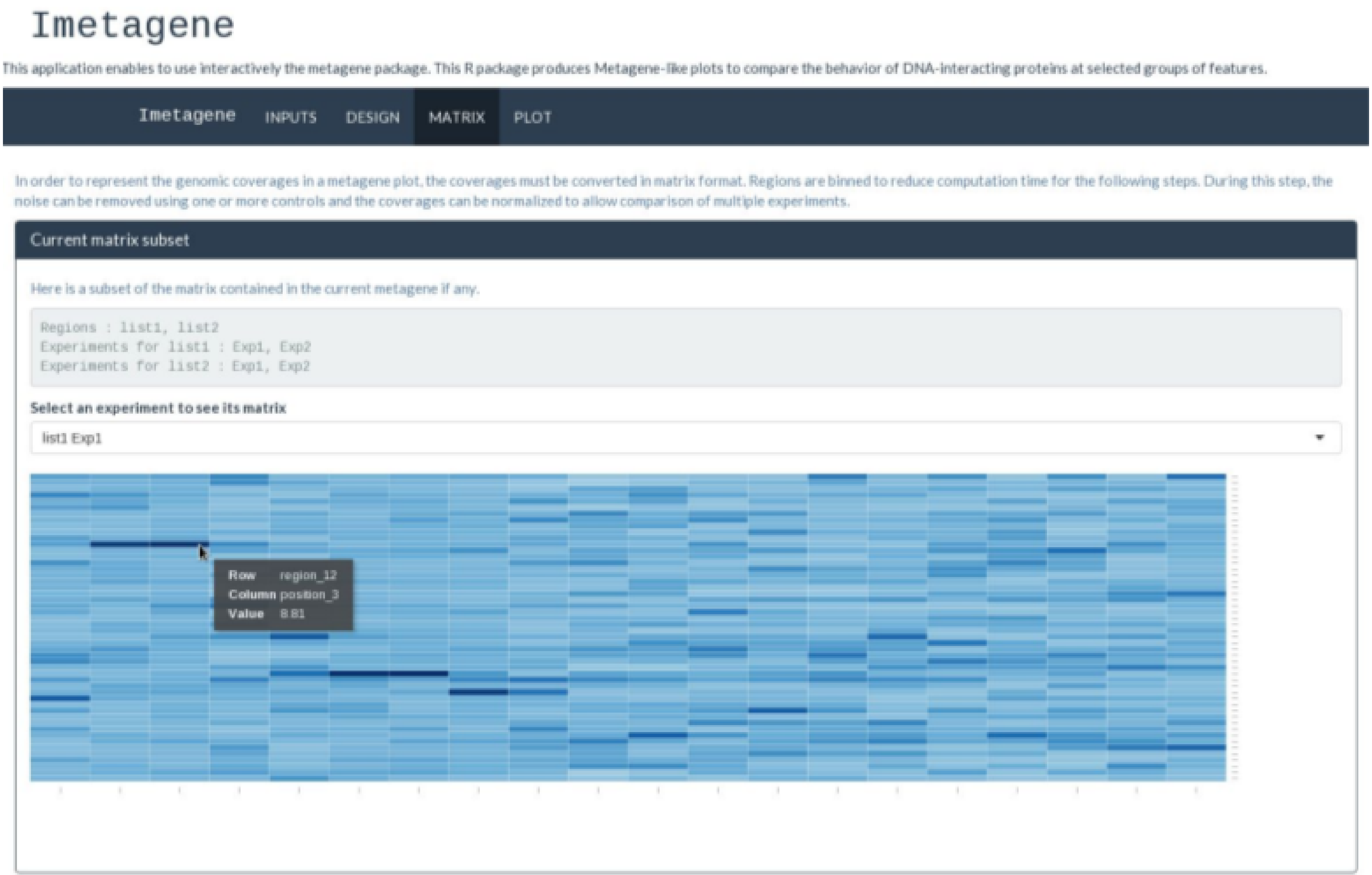

Supplement: S1 Fig — After the matrices are computed, the Imetagene package can be used to explore the matrix-associated with each experiment to visualize the coverages of the regions. (PDF) [file pcbi.1004751.s001.pdf]
