# Supplementary figures and images for: metagene Profiles Analyses Reveal Regulatory Element’s Factor-Specific Recruitment Patterns
Source: PLoS Comput Biol. 2016 Aug 18;12(8):e1004751. doi: 10.1371/journal.pcbi.1004751 (PMC4990179; doi:10.1371/journal.pcbi.1004751)

**S2 Fig. Metagene plots of POLR2AphosphoS2 in promoters and enhancers**

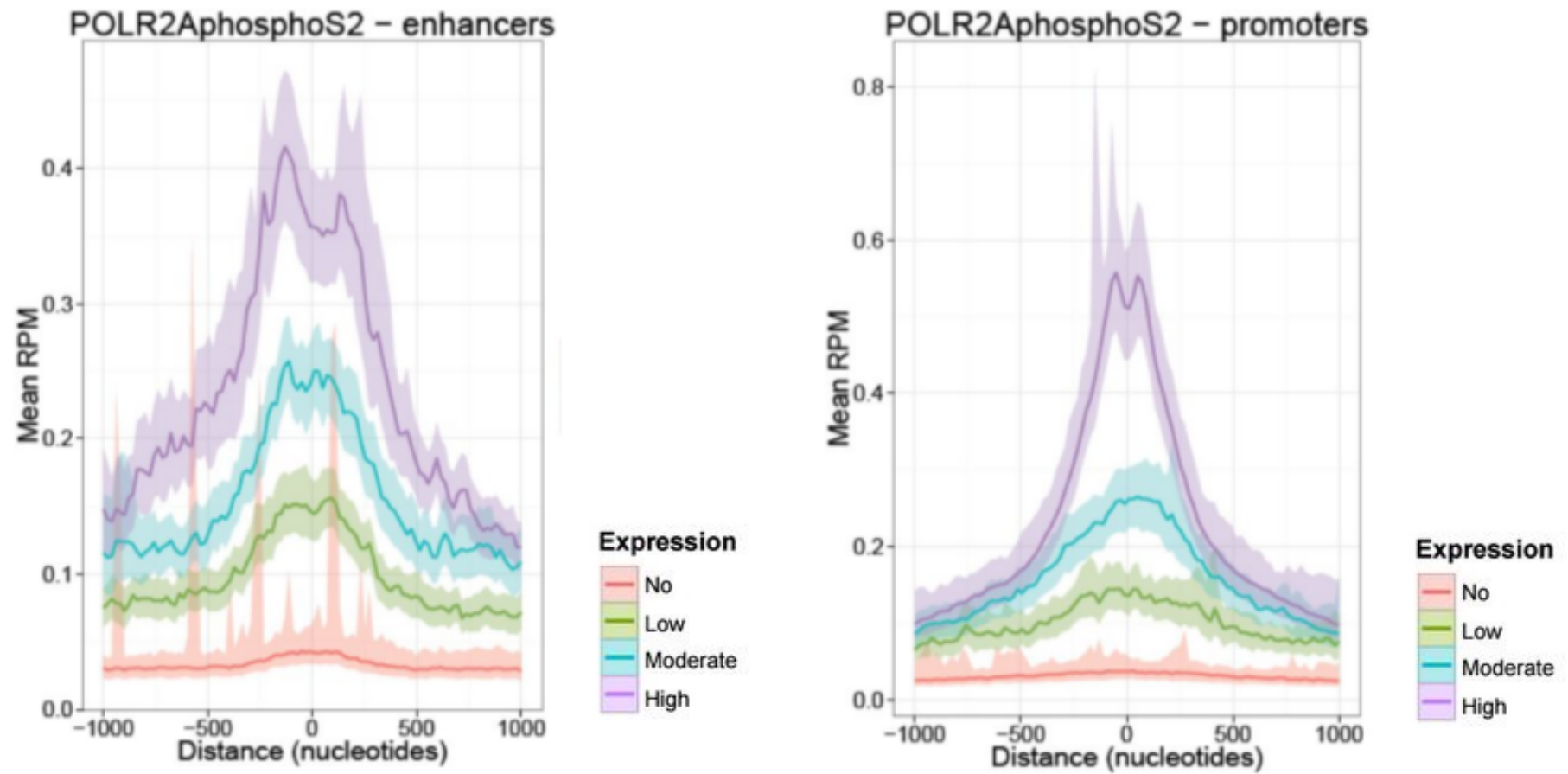

Supplement: S2 Fig — The x-axis is centered on enhancers and promoters ±1000bp. The y-axis represents the mean occupancy normalized in reads per million (RPM). Each line represents the mean occupancy of POLR2Aphosphos2. Groups of transcriptional activity of enhancers or promoters are identified by different colors (red = no CAGE signal; green = low CAGE signal; blue = moderate CAGE signal; purple = high CAGE signal; see S1 Text). Ribbons represent the 95% confidence interval of the mean calculated using 1000 bootstraps. (PDF) [file pcbi.1004751.s002.pdf]

**S3 Fig. Metagene plots of POLR2AphosphoS5**

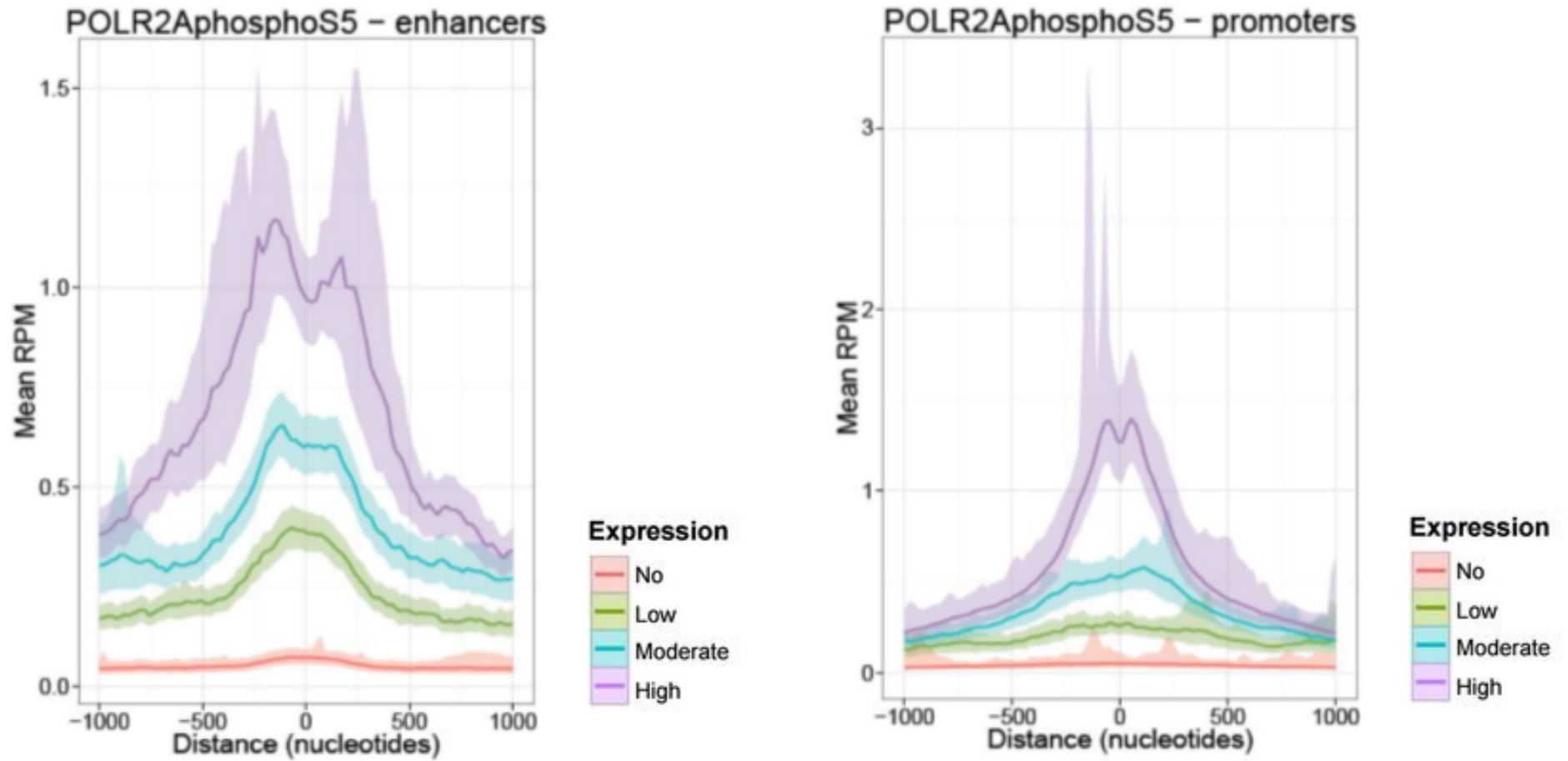

Supplement: S3 Fig — The x-axis is centered on enhancers and promoters ±1000bp. The y-axis represents the mean occupancy normalized in reads per million (RPM). Each line represents the mean occupancy of POLR2Aphosphos5. Groups of transcriptional activity of enhancers or promoters are identified by different colors (red = no CAGE signal; green = low CAGE signal; blue = moderate CAGE signal; purple = high CAGE signal; see S1 Text). Ribbons represent the 95% confidence interval of the mean calculated using 1000 bootstraps. (PDF) [file pcbi.1004751.s003.pdf]

**S4 Fig. Metagene plots of the general transcription factor TBP at promoters and enhancers**

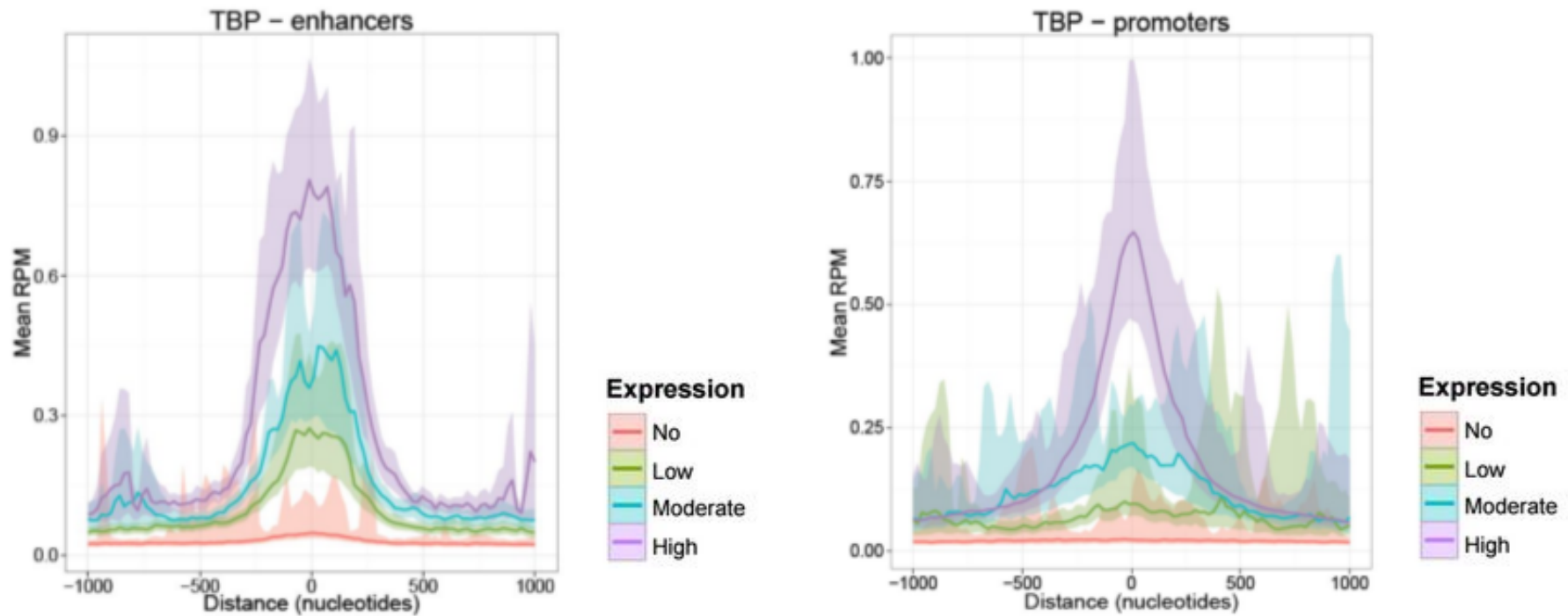

Supplement: S4 Fig — The x-axis is centered on enhancers and promoters ±1000bp. The y-axis represents the mean occupancy normalized in reads per million (RPM). Each line represents the mean occupancy of TBP. Groups of transcriptional activity of enhancers or promoters are identified by different colors (red = no CAGE signal; green = low CAGE signal; blue = moderate CAGE signal; purple = high CAGE signal). The ribbons represent the 95% confidence interval of the mean calculated using 1000 bootstraps. (PDF) [file pcbi.1004751.s004.pdf]

**S5 Fig. Metagene plots H3K27ac in enhancers and H3K4me3 at promoters**

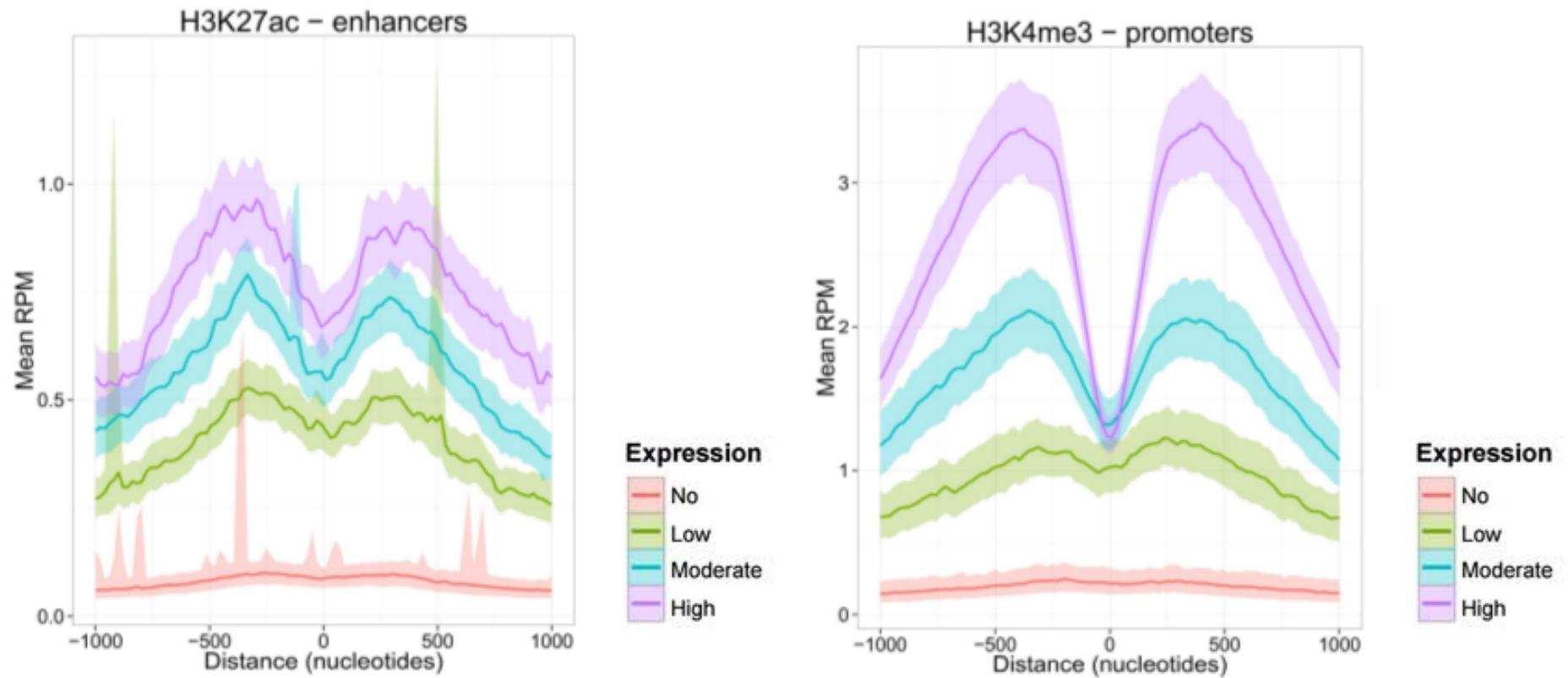

Supplement: S5 Fig — The x-axis is centered on enhancers and promoters ±1000bp. The y-axis represents the mean occupancy normalized in reads per million (RPM). Each line represents the mean occupancy of the histone mark. Groups of transcriptional activity of enhancers or promoters are identified by different colors (red = no CAGE signal; green = low CAGE signal; blue = moderate CAGE signal; purple = high CAGE signal). The ribbons represent the 95% confidence interval of the mean calculated using 1000 bootstraps. (PDF) [file pcbi.1004751.s005.pdf]

**S6 Fig. Boxplot of RATION\_INTERSECT values for 105 experiments in GM12878**

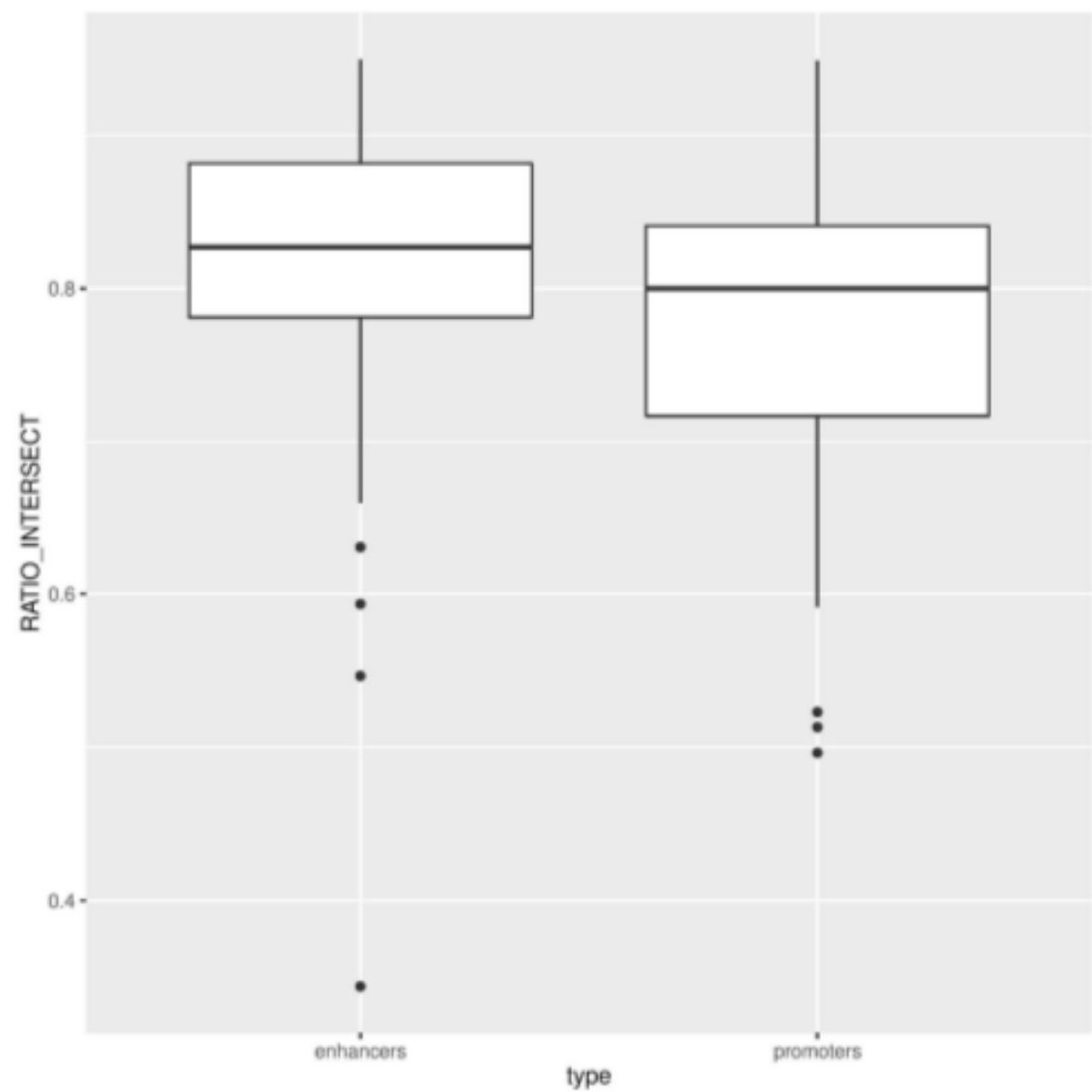

Supplement: S6 Fig — The RATIO INTERSECT was calculated using the moderate CAGE signal and high CAGE signal groups. (PDF) [file pcbi.1004751.s006.pdf]

**S7 Fig. Metagene plots of the cofactor SMC3 at promoters and enhancers**

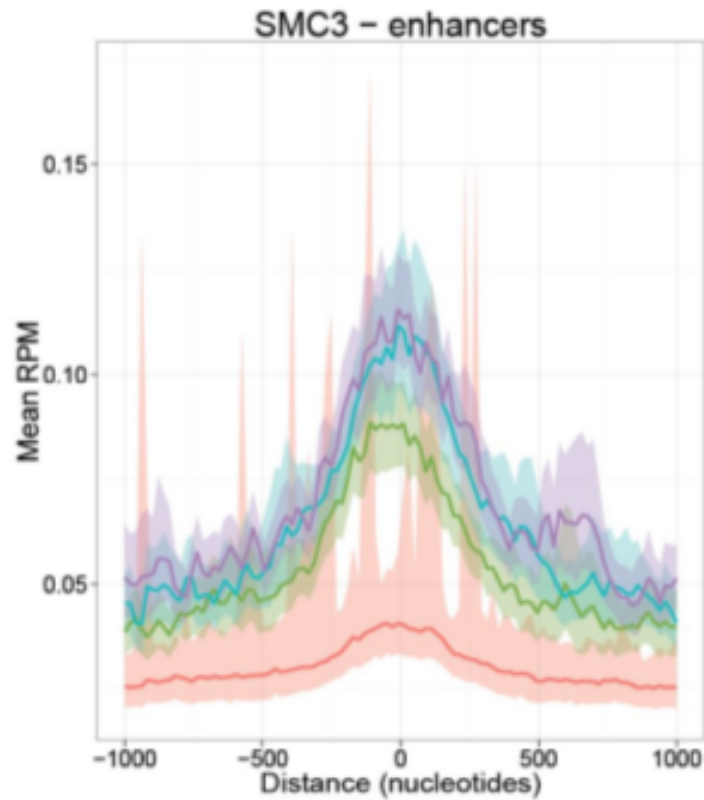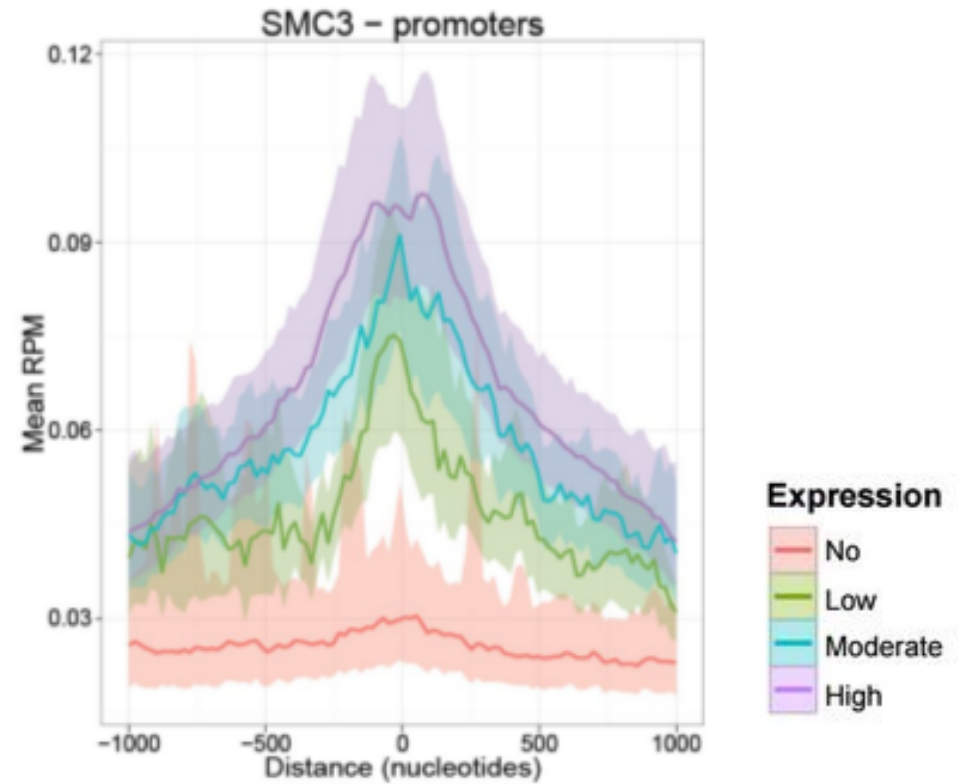

Supplement: S7 Fig — The x-axis is centered on enhancers and promoters ±1000bp. The y-axis represents the mean occupancy normalized in reads per million (RPM). Each line represents the mean occupancy of SMC3. Groups of transcriptional activity of enhancers or promoters are identified by different colors (red = no CAGE signal; green = low CAGE signal; blue = moderate CAGE signal; purple = high CAGE signal). The ribbons represent the 95% confidence interval of the mean calculated using 1000 bootstraps. (PDF) [file pcbi.1004751.s007.pdf]

**S8 Fig. Metagene plots of the cofactor EP300 at promoters and enhancers**

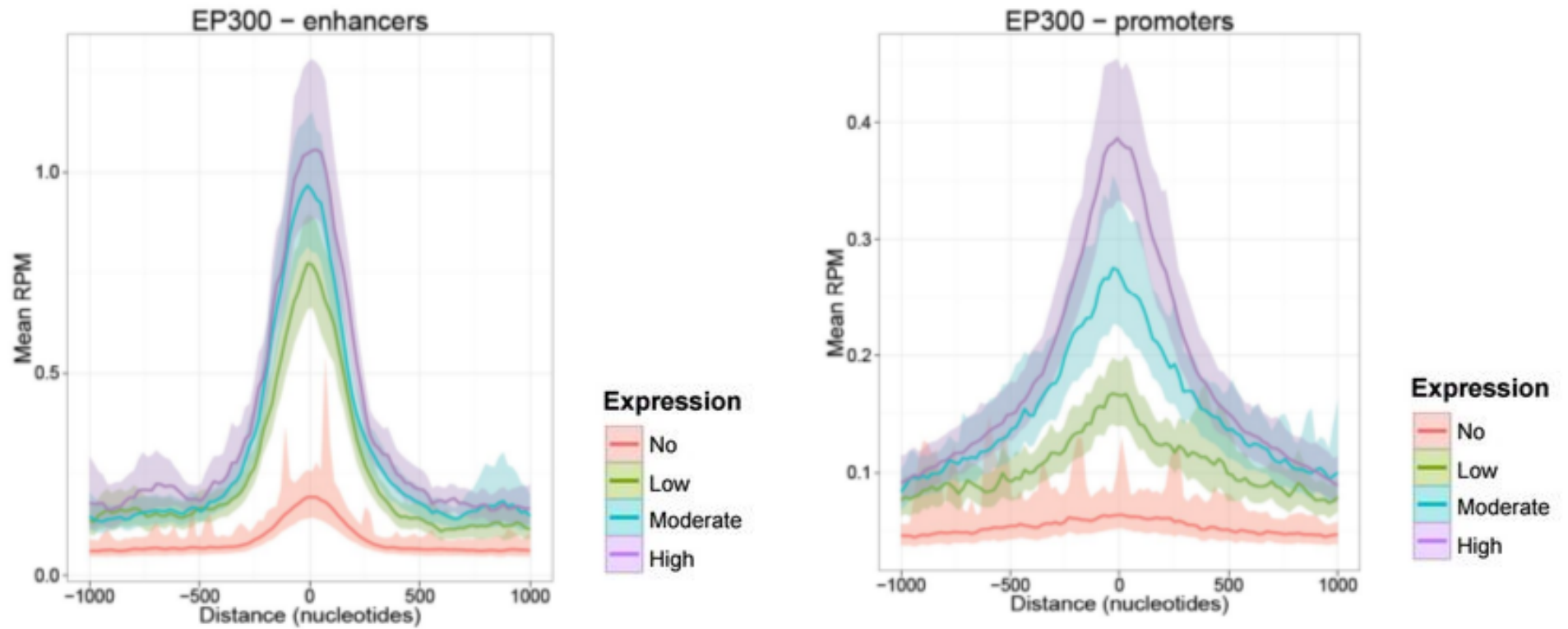

Supplement: S8 Fig — The x-axis is centered on enhancers and promoters ±1000bp. The y-axis represents the mean occupancy normalized in reads per million (RPM). Each line represents the mean occupancy of EP300. Groups of transcriptional activity of enhancers or promoters are identified by different colors (red = no CAGE signal; green = low CAGE signal; blue = moderate CAGE signal; purple = high CAGE signal). The ribbons represent the 95% confidence interval of the mean calculated using 1000 bootstraps. (PDF) [file pcbi.1004751.s008.pdf]
