# Supplementary material for: metagene Profiles Analyses Reveal Regulatory Element’s Factor-Specific Recruitment Patterns
Source: PLoS Comput Biol. 2016 Aug 18;12(8):e1004751. doi: 10.1371/journal.pcbi.1004751 (PMC4990179; doi:10.1371/journal.pcbi.1004751)
Supplement: S1 Text — Bootstrap: Description of the bootstrapping steps. Permutation: Details of the permutation procedure in Metagene and similaRpeak. (PDF) [file pcbi.1004751.s012.pdf]

S1 Text. *metagene* profiles analyses reveal regulatory elements specific recruitment pattern

Charles Joly Beauparlant[1,2,5], Fabien C. Lamaze[1,3,5], Astrid Deschênes[1], Rawane Samb[1], Audrey Lemaçon[1], Pascal Belleau[1], Steve Bilodeau[1,3,4] and Arnaud Droit[1,2,*]

[1]Centre de Recherche du CHU de Québec, Université Laval, Québec, Québec, G1V 0A6
[2]Département de Médecine Moléculaire, Faculté de médecine, Québec, Canada
[3]Centre de Recherche sur le Cancer de l'Université Laval, 9, rue McMahon, Québec, Québec, G1R 3S3
[4]Département de Biologie Moléculaire, Biochimie Médicale et Pathologie, Faculté de médecine, Québec, canada
[5]Co-first authors.

Data collection

First, we stratified four groups of enhancer and promoter regions based on their transcriptional levels using cap analysis of gene expression (CAGE) levels [28]. Transcription start sites (TSS) with a transcript per million (TPM) value greater than 0 were extracted from Fantom5 for the GM12878 and split into low expression (smaller or equal to the 33th percentile), moderate expression (between the 33th and 66th percentile) and high expression (greater than 66th percentile) groups. Regions with a TPM value of 0 were included into the "no expression" group. The Fantom consortium defines enhancers as regions with balanced bidirectional capped transcripts [28], as measured by CAGE. Fantom also defines a subset of TSS as 'robust' using a tag evidence thresholds approach [38]. Each enhancer region contains between 0 and 20 robust TSS. We studied enhancers from the Fantom 5 release that overlap at least one 'robust TSS' and we defined the expression level or the enhancer as the maximal TPM value of the overlapped TSS. Enhancers were resized to a final width of 2000 nucleotides to produce the robust enhancers regions. The promoters were defined using the Bioconductor's TxDb.Hsapiens.UCSC.hg19.knownGene package [16] with 1500 nucleotides upstream and 500 nucleotides downstream of each Entrez gene TSS position and were filtered to include only the regions overlapping at least one robust TSS. Their expression level was defined as for the enhancers. Each region was then centered on the Entrez gene TSS position. The files were downloaded with the ENCODExplorer package [39].

Bootstrap

The metagene package produces one curve for each combination of group of genomic regions and sample. To calculate the value of the points in the curve, metagene calculates the estimator (mean or median) value of each row in a matrix where columns correspond to a bin and each row to a genomic region. The bootstrapping step is performed to calculate the confidence interval (CI) on the estimator. The CI of each curve is calculated independently. For each bin position, metagene will sample with replacement the values. By default, metagene will produce 1000 samples (can be changed with the sample_count parameter of the produce_data_frame function) of the size of the smallest number of rows of all the matrices (can be changed with the sample_size parameter of the produce_data_frame function). The estimator value is computed for each sample and the result is used to calculate the 0.025 and 0.975 CI (alpha = 0.05).

Permutation

It is possible to compare two profiles using a metric of interest (see Metrics description section of this document for a list of recommended metrics implemented in the similaRpeak package). The metagene package implements a permutation strategy to determine if two profiles are

statistically different for a given metric. The permutation analysis will perform multiple iterations (the number of permutation can be defined with the sample_count parameter). For each iteration, two new profiles are produced by randomly sampling elements for the combination of the two matrices used to produce the original profiles. The number of element sampled for each profile is defined with the sample_size parameter. The metric value is then calculated using the new profiles. The ratio of metrics values with a score greater or equal to the original metric value can be used to compute a p-value and determine if the two original profiles are statistically different.

Supplementary Bibliography:

[38] Consortium TF, et al. A promoter-level mammalian expression atlas. Nature. 2014;507(7493):462–470.

[39] Beauparlant CJ, Lemacon A, Droit A. ENCODExplorer: A compilation of ENCODE metadata; 2015. R package version 1.2.1.
